# Optical Coherence Tomography Angiography: Investigating Vessel Density Changes Induced by Caffeine in Healthy Subjects

**DOI:** 10.1155/2024/5597188

**Published:** 2024-10-28

**Authors:** Mitchell Jacobs, Nicholas Demas, Angela Hemesath, Christopher Turski, Nicholas Fowler, John Benjamin Chadwell, Alec Dupont, Victoria Kupper, Kishor Acharya, Sarah Robbins, Kory Heier, Ramiro Maldonado

**Affiliations:** ^1^Department of Ophthalmology and Visual Sciences, University of Kentucky, Lexington, Kentucky, USA; ^2^Department Eye Center, Duke University, Durham, North Carolina, USA; ^3^Center for Clinical and Translational Service, University of Kentucky, Lexington, Kentucky, USA; ^4^Department of Biostatistics, University of Kentucky, Lexington, Kentucky, USA

## Abstract

**Introduction:** Caffeine, the most widely consumed psychoactive drug globally, has been associated with vascular changes in various organs, including the retina. Researchers have reported vascular constriction in the retina in response to caffeine, although data on its effects remain limited and somewhat contradictory. Further research is needed to clarify the specific impact of caffeine on retinal blood vessels and its potential implications for ocular health.

**Purpose:** To investigate the effects of 200 mg of caffeine on systolic and diastolic blood pressure (SBP and DBP) and retinal vessel density (VD) assessed by optical coherence tomography angiography (OCTA).

**Methods:** Prospective randomized, double-blind placebo-controlled, IRB-approved study in 59 healthy low caffeine users (< 136 mg of caffeine daily). Baseline 3 × 3 and 6 × 6 mm OCTA scans centered on the fovea as well as a 6 × 6 mm scans centered on the optic nerve head (ONH) were obtained. Participants were randomly assigned into caffeine group (CG, *n* = 42) receiving 200 mg caffeine pill or placebo group (PG, *n* = 17). OCTA scans were repeated at 60 and 120 min after intervention. VD was measured with Advanced Retina Imaging (ARI) network software (Carl Zeiss Meditec, Dublin, CA) for superficial capillary plexus (SCP) and deep capillary plexus (DCP). SBP/DBP readings were recorded before each imaging session. Ordinary one-way analysis of variance (ANOVA) of each group was performed using GraphPad Prism Version 9.3.0.

**Results:** Both groups had comparable demographics and OCTA parameters at baseline. Two hours after intervention, the CG had a significantly higher SBP (123 ± 7 mmHg) and DBP (81 ± 5 mmHg) compared to the control group (118 ± 7 mmHg, 77 ± 6 mmHg) (*p* value = 0.012, 0.023). Regarding the OCTA VD metrics, there were no significant differences in VD between the caffeine and placebo groups, regardless of whether the scans were centered on the macula or ONH. Additionally, the comparison across different OCTA scan modalities, specifically the 3 × 3 mm and 6 × 6 mm scans, showed no discernible differences among groups.

**Conclusion:** In conclusion, 200 mg of caffeine elevated blood pressure after 2 h but did not impact the retinal VD. This underscores the intricate relationship between caffeine, blood pressure, and retinal vascular dynamics, prompting further exploration of their implications for ocular health, especially in subjects with vascular disease.

## 1. Introduction

Caffeine, the world's most commonly used psychoactive drug, is consumed daily by 85% of the U.S. population [1–3]. Despite its widespread usage, there is still much to learn about its effects on the human body, making ongoing research crucial for a comprehensive understanding.

While the FDA recommends a daily limit of 400 mg of caffeine, even lower doses can influence vascular health [4–10]. Research presents conflicting findings, with some studies suggesting adverse effects on blood pressure and cardiovascular risk, while meta-analyses propose potential cardiovascular benefits [11–16]. Notably, caffeine's vascular and anti-inflammatory effects are associated with a 65% decreased risk of dementia and Alzheimer's disease in coffee consumers [17, 18]. The ongoing exploration of caffeine's intricate impact on vascular health continues to generate discussion and research in the scientific community.

Caffeine's impact on the eyes has been studied extensively, revealing temporary increases in intraocular pressure (IOP) and reduced blood flow in critical eye regions [19, 20]. Recent research, employing advanced imaging technology like optical coherence tomography angiography (OCTA), indicates a consistent finding: Caffeine leads to decreased vessel density, suggesting a vasoconstrictive effect on retinal circulation [21, 22].

OCTA, introduced clinically in 2006, is a powerful tool for visualizing retinal microvasculature with high resolution [23–29]. Despite susceptibility to motion and flow artifacts, it is well-suited for large-scale studies due to its rapid acquisition [30, 31]. With two main types, spectral-domain (SD) and swept-source (SS), we opt for SS-OCT for its faster scanning speed, providing improved scan patterns, wider field of view, and deeper penetration into retinal layers [32]. Our focus is on studying the effects of caffeine on retinal circulation, aiming to replicate and extend previous research by conducting assessments at 60 and 120 min for a thorough exploration of caffeine's impact on retinal blood flow.

## 2. Methods

### 2.1. Participants

This study was approved by the Institutional Review Board of the University of Kentucky and adhered to the Declaration of Helsinki. Written informed consent was obtained from each subject prior to their participation. A total of 59 healthy subjects were included in the study. All subjects were healthy individuals recruited through outreach tools provided by the University of Kentucky between October 2021 and March 2022.

### 2.2. Study Design

This was a prospective randomized, double-blind placebo-controlled study, taking place at the Department of Ophthalmology and Visual Sciences at the University of Kentucky.

Subjects were asked to abstain from any caffeine consumption 8 h prior to the study. Subjects underwent thorough eye examination prior to imaging to ensure no ocular pathology was present. Each participant was imaged between 8 am and 9 am to control for diurnal variations. Subjects were randomly assigned to the caffeine or control groups. Three baseline images were done bilaterally, and baseline blood pressure was measured. Subjects were then given either a 200 mg caffeine pill or placebo capsule containing 200 mg lactose powder. Caffeine pills were obtained from the University pharmacy. After capsule administration, bilateral OCTA and blood pressure measurements were repeated at an hour after capsule administration and then again 2 h after capsule administration. Only one eye was selected for analysis, although both eyes were imaged for all subjects. The intraclass correlation coefficient between the eyes exceeded 0.90 for the SCP and DCP metrics. Participants were instructed not to consume any food or beverages before or in-between imaging.

Exclusion criteria for all participants included the following: (1) any history or physical exam findings of ocular disease or trauma, including nystagmus, ocular surgeries, cataract, glaucoma, and retinopathies; (2) refractive spherical diopter > 6 D; (3) medical history of epilepsy or migraines; (4) systemic diseases affecting ocular microvasculature such as diabetes mellitus, hypertension, and renal or cardiovascular disease; (5) any chronic medication use affecting blood pressure including anti-hypertensives, analgesics, and antihistamines; (6) pregnant or nursing subjects; and (7) current or nonremote history of smoking.

### 2.3. Examination

At the start of the visit, participants reported their height and weight from which body mass index (BMI) was calculated. Demographic information including age, sex, smoking history, average daily caffeine consumption, and medical history was also collected. Subjects were further divided into either low caffeine (< 130 mg daily) or high caffeine (> 130 mg daily) users. SBP and DBP were measured at the brachial artery using an automatic blood pressure cuff. Refraction and axial length were measured with an autorefractor, or lensometer if the subject used glasses. Best-corrected visual acuity (VA) was collected bilaterally for each participant using standard techniques on a Snellen chart. IOP was measured bilaterally using indentation tonometry with a Tono-Pen tonometer (AMETEK Reichert Technologies, Buffalo, NY).

### 2.4. OCTA Measurements

Images were acquired using the ZEISS PLEX Elite 9000 SS-OCTA (Carl Zeiss Meditec, Dublin, CA), which has dual-speed scanning at 100,000 and 200,000 A-scans/sec, A-scan depth of 3.0 or 6.0 mm, and a tunable laser between 1040 and 1070 nm. SS-OCT uses a broadband swept source light wavelength that varies with time, as opposed to SD-OCT which obtains its interference spectrum through spectral splitting. As a result, SS-OCT has a wide range of scanning and fast scan rate. Although the swept source technology is faster than SD-OCT, it does have a relatively lower definition.

Images were analyzed with the instrument's ARI network software (Carl Zeiss Meditec, Dublin, CA) superficial perfusion density algorithm for VD defined as the total area of perfused vasculature in a region of measurement. Baseline 3 × 3 mm and 6 × 6 mm OCTA scans centered over the fovea and 6 × 6 mm scans centered over the ONH were obtained. Automatic segmentation by the software was done to measure VD in the SCP and DCP.

The macular superficial VD (mm) was measured between the inner limiting membrane and the inner plexiform layer. The macular deep VD (mm) was measured between the inner plexiform layer and the outer plexiform layer.

### 2.5. Statistical Methods

Demographic and ocular characteristics of treatment groups were compared with independent *t-*tests and *χ*^2^ tests for continuous and categorical variables, respectively. One eye from each subject was chosen randomly for statistical analysis. Mixed-effects regression models assessed the ocular response to caffeine using the lmerTest *R* package (version 3.1–3). Three-way interactions between dose group (caffeine vs. placebo), user type (high caffeine user vs. low caffeine user), and time (hours) were included with random intercepts for each subject to account for the correlation that exists between ocular response of that same subject. The average of the first three baseline measurements for each participant was taken. Results were reported using a Type III ANOVA table with the following criteria: (1) if the three-way interaction fails to reach significance, all two-way interactions will be tested; (2) if the two-way interaction fails to reach significance, they will be removed, and only the main effects will be reported. *p* values of the ANOVA table were computed using Satterthwaite's method. Comparisons of baseline OCTA measurements and one-hour and two-hour measurements were done for the high caffeine user case group, the high caffeine user control group, the low caffeine user case group, and the low caffeine user control group using Dunnett's multiple comparisons test. *p* values < 0.05 were determined to be statistically significant. Statistical analyses were performed through *R* and *R* Studio software (R Foundation for Statistical Computing, Vienna, Austria, https://www.R-project.org/).

## 3. Results

Study participant demographics are listed in Table 1. No study participants reported a history of hypertension, diabetes, or medications that are used for vascular diseases. There were no significant differences in demographic information between the control group and the experimental group. The mean age of the study group was 23 (range of 18.3–31.5) and the mean age of the PG was 23.2 (range of 18.6–34). The study group had 69% female subjects, and the control group had 64% female subjects. The average caffeine use was 188 mg/day in the CG and 186 mg/day in the control group. Average BMI in the CG was 24.1 kg/m^2^ and 25.2 kg/m^2^ in the control group. Baseline SBP was 122 mmHg in the CG and was 119 mmHg in the control group. There was a significant difference between the average DBP in the caffeine and control groups at 81.7 mmHg and 77.4 mmHg (*p*=0.0178), respectively. IOP and ocular axial lengths were not different between groups (Table 1). The OCTA variables were no different between groups at baseline (Table 2).

One hour after intervention, there was no difference in SBP or DBP between the two groups. However, after two hours, the CG had a significantly higher SBP (123 ± 7 mmHg) and DBP (81 ± 5 mmHg) compared to the control group (118 ± 7 mmHg, 77 ± 6 mmHg) (*p* value = 0.012, 0.023) (Table 3). Additionally, the treatment group had a significant rise in SBP from baseline to 1 h after intervention (*p*=0.005), as well as from baseline to 2 h after intervention (*p* value = 0.025). There were no significant differences in SBP or DBP in the control group one or two hours after intervention.

**Figure 1:**
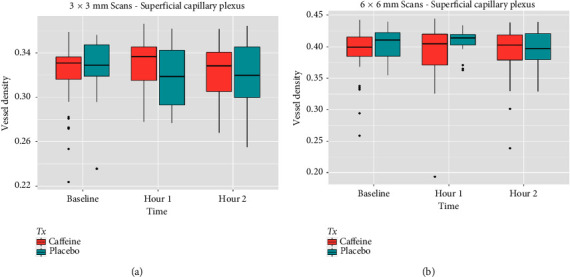
Vessel density box plots from *superficial capillary plexus* from 3 × 3 mm (a) and 6 × 6 mm (b) OCTA scans indicate no significant differences between caffeine and placebo groups at baseline, 1 h, and 2 h. Both groups show similar trends in central tendencies and variability over time.

**Figure 2:**
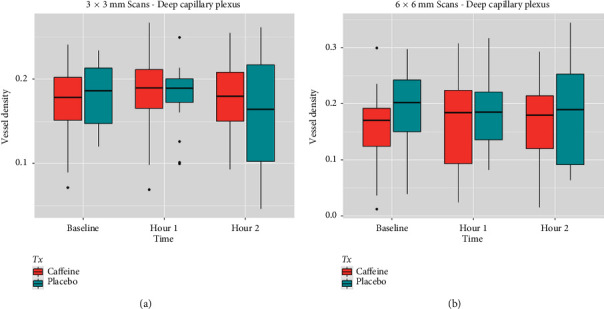
Vessel density box plots from *deep capillary plexus* from 3 × 3 mm (a) and 6 × 6 mm (b) OCTA scans indicate no significant differences between caffeine and placebo groups at baseline, 1 h, and 2 h. Both groups show similar trends in central tendencies and variability over time.

**Figure 3:**
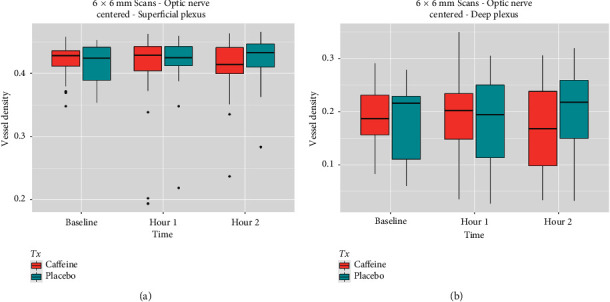
Vessel density box plots from *superficial and deep capillary plexus from scans centered at the optic nerve head* from 3 × 3 mm (a) and 6 × 6 mm (b) OCTA scans indicate no significant differences between caffeine and placebo groups at baseline, 1 h, and 2 h. Both groups show similar trends in central tendencies and variability over time.

One hour after drug administration, no significant difference in OCTA parameters was seen between the case group and the control group (Table 4 and Figures 1, 2, and 3). Similarly, two hours following capsule ingestions, there were no significant differences between the control and case groups (Table 4 and Figures 1, 2, and 3).

Furthermore, there were no differences in OCTA parameters at either one hour or two hours after intervention for high caffeine users or low caffeine users between the control group and case group (Tables 5 and 6).

In the low caffeine user case group, there were no significant differences in OCTA parameters between the average 3 baseline measurements and the follow-up images one hour later. Similarly, no significant differences were seen from the baseline measurements and the two-hour follow-up measurements (Table 7).

No significant difference was seen in OCTA measurements within the low caffeine user control group at baseline vs. 1 h follow-up and baseline vs. 2 h follow-up (Table 7).

No significant difference in 3 × 3 mm DCP, 6 × 6 DCP, 6 × 6 superficial, ONH DCP, and ONH superficial measurements were seen in the high caffeine user treatment group both at baseline and 1 h follow-up and baseline and 2 h follow-up. No difference was seen in the 3 × 3 superficial measurement at baseline vs. 1 h follow-up. A significant difference in 3 × 3 superficial VD was seen when comparing baseline vs. the 2 h follow-up (*p*=0.045) (Table 7).

No significant differences in OCTA parameters were seen in the high caffeine user PG when comparing baseline measurements to 1 h follow-up measurements. Similarly, there were no significant differences in OCTA parameters when comparing baseline measurements to 2 h follow-up measurements (Table 7).

## 4. Discussion

The findings of this study suggest that caffeine did not influence the VD of the macular or peripapillary regions of the retina, but other factors may play a role in previously documented vasoconstrictive effects. Throughout all but one assessed time point, there were no notable discrepancies observed in OCTA measurements among healthy individuals following caffeine consumption. Of note, there was a statistically significant difference in the 3 × 3 superficial VD when comparing baseline vs. the 2 h follow-up measurement, only in high-caffeine users (Table 7). No other discernible distinctions in OCTA measurements were evident between case and control subjects at baseline, as well as at 1 h and 2 h intervals post-intervention.

These findings diverge from previous research, such as the study conducted by Tugan et al. [33], which revealed a notable reduction in superficial, deep, and peripapillary VD 1 h following the ingestion of 200 mg of caffeine. Their study, encompassing 120 subjects and employing a study design akin to ours, utilized a different OCTA system (OptoVue-AngioVue), characterized by slower imaging technology (SD-OCT at 70,000 A-scans per second), as opposed to our OCTA system (ElitePlex-Zeiss at 400,000 A-scans per second). Comparable to our approach, they employed a 6 × 6 mm scan size.

Upon scrutinizing their findings, the discrepancy noted at the one-hour mark proves statistically significant; however, the medians between baseline and post-caffeine intake differ within a range of merely one to three percentage points for most parameters. This marginal variance, though potentially reflective of caffeine's effect, is arguably too minute to serve as a robust indicator in clinical practice.

OCTA technology is susceptible to various artifacts and subsequent changes in variability. For example, Tugan et al. [33], showed noticeable areas of flow void voids, which is not typically expected in a healthy retina, yet post-caffeine intake, these areas appear to exhibit flow, suggesting a potential improvement in circulation due to caffeine. This is a controversial statement and underscores a significant drawback of OCTA technology, which is susceptible to various artifacts and session-to-session variability. Furthermore, in this particular example from Tugan et al. [33], it is apparent that VD metrics may have been influenced by errors in vessel projection removal, as vessel shadowing seems inconsistently addressed between the pre- and post-caffeine scans. Our own study scans also reveal challenges in effectively removing vessel projection artifacts through algorithms. Consequently, VD metrics at the DCP may be regarded as a less reliable marker, given its reliance on an imperfect algorithm.

Another study, with comparable design objectives, is conducted by Karti et al. [34]. Their research showcased noteworthy reductions in VD within both the SCP and DCP at various retinal locations, including the fovea, parafovea, and all four quadrants, 1 h following the ingestion of 200 mg of caffeine compared to a PG. Employing the OptoVue OCTA system and utilizing 6 × 6 mm scans, Karti et al. [34] also corroborated diminished VD one-hour post-caffeine consumption. Once again, while the outcomes prove statistically significant, the mean disparities between the placebo and caffeine cohorts typically range within one to two percentage points.

Our study employed SS-OCTA technology, which boasts a faster scanning speed compared to the SD-OCT utilized in previous investigations. Notably, SS-OCTA has not been previously utilized in studies exploring the effects of caffeine on retinal microvasculature. Our rationale was to assess the reproducibility of prior findings using a swifter technology, known to be less susceptible to motion artifacts. This is particularly pertinent given studies such as the one conducted by Weist et al. [35], which reported significant variations in VD values measured by different imaging systems and VD calculation algorithms [33]. In an ideal scenario, findings observed with a slower imaging technology should be corroborated with a faster and higher resolution technology [34].

Interestingly, other researchers have undertaken a similar study design, albeit with flavanol-rich dark chocolate, which is believed to induce nitric oxide-dependent vasodilation and lower systemic blood pressure [36]. In their experiment involving 22 healthy participants, they utilized the OptoVue system and performed 6 × 6 mm OCTA scans. However, they did not observe any disparity in OCTA outcome markers between the treatment and placebo groups, suggesting contrasting effects compared to caffeine [36]. Other investigations have also yielded divergent findings regarding changes in retinal VD. For instance, Shoeibi et al. [37], employing OptoVue OCTA scans measuring 3.5 mm × 3.5 mm, noted a reduction in superficial and deep parafoveal VD following the ingestion of 130 mg of caffeine, albeit with no alterations detected in the perifoveal or peripapillary regions after 45 min. Additionally, Zhu et al. [38] observed increased retina-choroid blood flow and a decrease in superficial retinal layer vessel diameter index (VDI) two hours post-consumption of 72 mg of caffeine, using Cirrus-OCTA technology based on SD-OCT (68,000 A-scans per second). Law and Lam [39], in a study involving otherwise healthy high myopes administered with 200 mg of caffeine, reported a small yet significant decrease in VD after 180 min of intake. Huang et al. [30], investigating the effects of 200 mg of caffeine ingestion on 13 eyes, observed decreased SCD VD but increased DCP VD 50 s post-transition from dark to ambient light. However, one hour after caffeine intake (15 min post-light adaptation), DCP VD exhibited a significant decrease compared to controls [40]. Interestingly, this elevation in DCP VD during darkness contradicts the findings of Tugan et al. and Karti et al., who reported a significant decrease in VD across all regions [33, 34]. Similarly, Terai et al. [41] found that caffeine intake led to a reduction in the mean diameter of retinal arterioles, subsequently inducing vasoconstriction, increased flicker response, and elevated mean arterial pressure (MAP) following flicker light stimulation [39]. These discordant outcomes mirror the variability observed in the effects of caffeine on systemic vasculature.

Significantly, our study findings unequivocally showed a discernible rise in both systolic blood pressure and DBP following caffeine administration. Furthermore, no notable variations in demographics or ocular examination findings were observed between the two groups at baseline. This strongly implies that the outcomes observed in our study were not attributable to either a lack of caffeine efficacy or inherent dissimilarities between the case and control cohorts.

The alterations in retinal function hold significant implications within the realm of ocular pathology. For instance, in a study involving mice subjected to ischemia-reperfusion injury, topical application of caffeine led to the preservation of brain-derived growth factor levels and a decrease in IL-6 mRNA expression, indicating caffeine's potential to mitigate inflammation in retinal diseases [42]. Moreover, research conducted by Madeira et al. demonstrated that caffeine administration to rats afflicted with ocular hypertension yielded a reduction in neuroinflammatory responses of retinal microglia, further suggesting caffeine's therapeutic promise in retinal conditions [43].

The OCTA system (ElitePlex-Zeiss) has shown reliability in measuring VD and vascular changes and thus was deemed an appropriate and reliable marker for analyzing VD [44, 45].

Our study was intentionally designed with unequal randomization, favoring a significantly larger sample size for case subjects over controls, to ensure robust findings. However, this approach inevitably compromises the precision of the control group due to its comparatively smaller size, elevating the risk of type II error. Notably, our data analysis revealed no consistent pattern of larger standard deviations in control groups compared to treatment groups, thereby mitigating the likelihood of type II errors. Furthermore, it is important to acknowledge the limitations of our study, as we only assessed the average VD of the fovea and ONH at superficial and deep layers. Areas within the ETDRS circle and specific quadrants such as nasal, temporal, superior, and inferior were not evaluated, thus potentially overlooking relevant changes in these regions. Considering the inconsistent findings across studies employing comparable methodologies, alongside disparities between these findings and our own study, it is apparent that further investigation is warranted to elucidate the impact of caffeine on retinal circulation. Moreover, it is worth noting that OCTA might not be optimally suited for detecting subtle alterations in vasculature, given its susceptibility to artifacts. Furthermore, the clinical relevance of caffeine's purported effects, or the absence thereof, necessitates evaluation to enhance guidance for patients with ocular conditions like diabetic retinopathy. Our findings demonstrating no notable discrepancies in OCTA measurements using SS-OCTA may be due to factors unrelated to variations in blood pressure–related autoregulation. This may include the relatively younger groups of study participants as stated above with the correlating ability to better respond to changes in blood pressure, and the lack of any vascular-related chronic medical conditions, such as diabetes and hypertension, that would alter vasoconstrictive effects [46].

## Figures and Tables

**Table 1:** Caffeine and placebo groups' demographic, blood pressure, and ocular biometric characteristics.

	Caffeine group (*N* = 42)	Placebo group (*N* = 17)	*p*
*Age*			
Mean (SD)	23.0 (3.66)	23.2 (3.62)	0.836
Median [min, max]	21.7 [18.3, 31.5]	22.9 [18.6, 34.0]	

*Gender*			
(F/M)	29/13	11/6	0.988

*BMI*			
Mean (SD)	24.1 (3.19)	25.2 (4.84)	0.413
Median [min, max]	23.8 [18.3, 32.8]	23.7 [18.9, 35.5]	

*Caffeine use (mg per day)*			
Mean (SD)	188 (138)	186 (200)	0.971
Median [min, max]	147 [0, 502]	129 [0, 690]	

*Systolic BP (mmHg)*			
Mean (SD)	122 (7.57)	119 (8.07)	0.212
Median [min, max]	122 [102, 145]	118 [103, 132]	

*Diastolic BP (mmHg)*			
Mean (SD)	81.7 (6.56)	77.4 (5.81)	0.0178
Median [min, max]	82.0 [68.0, 100]	78.0 [68.0, 86.0]	

*OD IOP (mmHg)*			
Mean (SD)	16.3 (2.78)	16.1 (3.45)	0.811
Median [min, max]	16.5 [8.00, 21.0]	16.0 [9.00, 21.0]	

*OS IOP (mmHg)*			
Mean (SD)	16.1 (2.82)	15.8 (2.51)	0.617
Median [min, max]	16.0 [8.00, 22.0]	16.0 [12.0, 19.0]	

*OD axial length (mm)*			
Mean (SD)	23.8 (1.24)	23.3 (1.07)	0.129
Median [min, max]	23.8 [20.0, 27.2]	22.9 [21.7, 26.1]	

*OS axial length (mm)*			
Mean (SD)	23.8 (1.08)	23.4 (1.32)	0.273
Median [min, max]	24.1 [21.9, 25.9]	23.3 [21.5, 27.1]	

**Table 2:** Caffeine and placebo groups' OCTA vessel density variables at baseline.

	Caffeine group (*N* = 42)	Placebo group (*N* = 17)	*p*
*Baseline superficial 3 mm*			
Mean (SD)	0.321 (0.0284)	0.327 (0.0293)	0.477
Median [min, max]	0.331 [0.224, 0.359]	0.333 [0.235, 0.356]	

*Baseline superficial 6 mm*			
Mean (SD)	0.392 (0.0363)	0.404 (0.0270)	0.167
Median [min, max]	0.399 [0.259, 0.442]	0.411 [0.355, 0.439]	

*Baseline deep 3 mm*			
Mean (SD)	0.172 (0.0397)	0.182 (0.0380)	0.365
Median [min, max]	0.178 [0.0712, 0.241]	0.187 [0.120, 0.234]	

*Baseline deep 6 mm*			
Mean (SD)	0.158 (0.0573)	0.191 (0.0701)	0.0903
Median [min, max]	0.169 [0.0122, 0.300]	0.202 [0.0384, 0.297]	

*Baseline superficial optic nerve head*			
Mean (SD)	0.421 (0.0241)	0.415 (0.0334)	0.492
Median [min, max]	0.430 [0.348, 0.458]	0.424 [0.354, 0.453]	

*Baseline deep optic nerve head*			
Mean (SD)	0.190 (0.0500)	0.184 (0.0694)	0.74
Median [min, max]	0.187 [0.0823, 0.291]	0.209 [0.0596, 0.278]	

**Table 3:** Systolic and diastolic blood pressure in caffeine and control groups 1 h and 2 h after intervention.

		Caffeine group		Placebo group		*p*
		Mean (mmHg)	Standard deviation	Mean (mmHg)	Standard deviation	
Blood pressure comparison: caffeine group vs. placebo						
1 h follow-up	Systolic	123	8	119	8	0.090
	Diastolic	81	7	78	6	0.123
2 h follow-up	Systolic	123	7	118	7	0.012
	Diastolic	81	5	77	6	0.023

*Note*: *p* values indicate statistical significance (*p* < 0.05).

**Table 4:** OCTA variables among caffeine and placebo groups 1 h and 2 h after intervention.

	Caffeine group		Placebo group		*p*
	Mean (mm)	Standard deviation	Mean (mm)	Standard deviation	
*1 h mark*					
3 × 3 superficial	0.224	0.095	0.203	0.098	0.460
3 × 3 deep	0.183	0.043	0.179	0.040	0.760
6 × 6 superficial	0.391	0.044	0.407	0.022	0.175
6 × 6 deep	0.162	0.078	0.189	0.075	0.242
ONH superficial	0.413	0.054	0.413	0.058	0.915
ONH deep	0.189	0.074	0.189	0.090	0.846

*2* *h mark*					
3 × 3 superficial	0.225	0.095	0.223	0.086	0.961
3 × 3 deep	0.176	0.045	0.163	0.070	0.410
6 × 6 superficial	0.391	0.041	0.397	0.030	0.544
6 × 6 deep	0.162	0.073	0.181	0.088	0.398
ONH superficial	0.413	0.040	0.413	0.044	0.552
ONH deep	0.172	0.078	0.172	0.081	0.259

**Table 5:** OCTA variables among high caffeine users in control and treatment groups 1 h and 2 h after intervention.

	Caffeine group		Placebo group		*p*
	Mean (mm)	Standard deviation	Mean (mm)	Standard deviation	
*OCTA comparison at 1 h follow-up in high caffeine users*					
3 × 3 superficial	0.250	0.081	0.202	0.110	0.220
3 × 3 deep	0.177	0.046	0.182	0.045	0.810
6 × 6 superficial	0.394	0.035	0.412	0.025	0.242
6 × 6 deep	0.156	0.078	0.214	0.081	0.102
ONH superficial	0.429	0.031	0.401	0.082	0.189
ONH deep	0.201	0.063	0.186	0.095	0.636

*OCTA comparison at 2* *h follow-up in high caffeine users*					
3 × 3 superficial	0.217	0.106	0.208	0.112	0.840
3 × 3 deep	0.172	0.044	0.162	0.070	0.644
6 × 6 superficial	0.393	0.036	0.410	0.028	0.261
6 × 6 deep	0.163	0.072	0.201	0.101	0.281
ONH superficial	0.411	0.033	0.408	0.063	0.848
ONH deep	0.158	0.081	0.178	0.097	0.585

**Table 6:** OCTA variables among low caffeine users in control and treatment groups 1 h and 2 h after intervention.

	Caffeine group		Placebo group		*p*
	Mean (mm)	Standard deviation	Mean (mm)	Standard deviation	
*OCTA comparison at 1* *h follow-up in low caffeine users*					
3 × 3 superficial	0.197	0.102	0.205	0.094	0.853
3 × 3 deep	0.189	0.040	0.177	0.038	0.450
6 × 6 superficial	0.387	0.053	0.403	0.021	0.392
6 × 6 deep	0.169	0.079	0.170	0.068	0.983
ONH superficial	0.396	0.067	0.419	0.034	0.347
ONH deep	0.177	0.084	0.183	0.093	0.848

*OCTA comparison at 2* *h follow-up in low caffeine users*					
3 × 3 superficial	0.233	0.083	0.236	0.064	0.924
3 × 3 deep	0.180	0.046	0.165	0.074	0.482
6 × 6 superficial	0.388	0.047	0.388	0.029	0.976
6 × 6 deep	0.161	0.075	0.166	0.079	0.874
ONH superficial	0.415	0.047	0.430	0.022	0.367
ONH deep	0.187	0.073	0.214	0.067	0.353

**Table 7:** *p* values from OCTA measurements in low and high caffeine users at baseline, 1 h, and 2 h marks.

	Caffeine group		Placebo group	
	Baseline vs. 1 h (*p*)	Baseline vs. 2 h (*p*)	Baseline vs. 1 h (*p*)	Baseline vs. 2 h (*p*)
*Low caffeine users 0–2* *h OCTA comparison*				
3 × 3 superficial	0.2175	0.5124	0.3212	0.9208
3 × 3 deep	0.0697	0.4025	0.8499	0.7052
6 × 6 superficial	0.9773	0.9569	0.5281	0.6129
6 × 6 deep	0.702	0.6117	0.9329	0.4644
ONH superficial	0.3699	0.8001	0.8249	0.7299
ONH deep	0.2717	0.9302	0.9995	0.4306

*High caffeine users 0–2 h OCTA comparison*				
3 × 3 superficial	0.9787	0.045	0.9646	0.9992
3 × 3 deep	0.9697	0.7398	0.8616	0.8279
6 × 6 superficial	0.9727	0.7849	0.6404	0.8902
6 × 6 deep	0.8361	0.9047	0.4232	0.5783
ONH superficial	0.2972	0.2848	0.7182	0.9407
ONH deep	0.7111	0.1392	0.9414	0.9993

*Note:p* values were derived from Dunnett's multiple comparisons test.

## Data Availability

Data are available upon request. Contact Dr. Ramiro Maldonado for data requests.
